# The Search for the Genes of Vasovagal Syncope

**DOI:** 10.3389/fcvm.2019.00175

**Published:** 2019-11-28

**Authors:** Robert S. Sheldon, Roopinder K. Sandhu

**Affiliations:** ^1^The Libin Cardiovascular Institute of Alberta, University of Calgary, Calgary, AB, Canada; ^2^The Mazankowski Heart Institute, University of Alberta, Edmonton, AB, Canada

**Keywords:** vasovagal syncope, genetics, kindreds, candidate gene, genome-wide association, syncope, vasovagal

## Abstract

Only humans faint, and not all do so. Syncope tends to recur, and the predisposition to syncope can persist over many decades. Observations such as these have suggested that there may be a genetic predisposition to vasovagal syncope. It seems to have a high prevalence in some families; having a parent who faints increases the likelihood of an offspring fainting, and this is increased even further if both biological parents faint. Numerous studies have correlated a number of genotypes with positive tilt tests. However, the control subjects are usually those who faint, but have negative tilt tests, making the conclusions about association with the clinical phenotype less certain. Twin studies, highly focused genome-wide association studies, and gene duplicate studies all suggest there are sites in the genome that associate with vasovagal syncope, although the specific genes, pathways, and proteins are unknown. A recent large, candidate gene study of kindreds with high, multigenerational prevalence of the vasovagal syncope identified 3 genes that associate with vasovagal syncope. Our understanding of the genetic correlates of vasovagal syncope is in its infancy, with much to be understood.

## Introduction

There are several reasons to suspect a genetic origin of vasovagal syncope. First, this appears to be a uniquely human response. First, there are no animals that faint (1), including closely related great apes. Second, not everyone faints. In countries such as Canada (2), the Netherlands (3), and Malaysia (4) there are similar proportions of lifetime syncope cumulative incidence, in the range of 25–35%. Therefore, only a proportion of people appear to have vasovagal syncope. Third, the predisposition to syncope lasts decades for many people and the predisposition to syncope is an enduring phenotypic trait. Most fainters start fainting by age 30, and many clinical studies report syncope recurrences over subsequent decades (5). Similarly, the predisposition to faint on tilt table testing is generally reproducible (6). Similarly, non-inducibility is also stable. Furthermore, the degree of bradycardia induced by tilt testing is reproducible (6). Lastly, yet less persuasively, vasovagal syncope has no clearly associated autoimmune or infectious etiology.

Taken together, these 4 factors—the absence of syncope in other animals, only an affected minority of the population, persistent clinical, and physiologic phenotypes, and no apparent other cause—suggest a genetic origin for vasovagal syncope in humans.

More direct evidence for genetic associations with vasovagal syncope is accumulating. The essence of genetic analysis is to test the statistical strength of the association with the phenotype compared to a control population. These methods studying families with fainting members, testing the association of pre-specified candidate genes with the phenotype, and performing genome-wide association studies. These different methodologies can be applied in kindred studies, conceptually related syndromes, and population-wide studies. Most reports of vasovagal syncope have been candidate gene studies of the surrogate outcome of fainting on a tilt test. Uncovering genetic associations with vasovagal syncope may provide insights into the physiology of the reflex, and perhaps new targets for therapy. We'll review the evidence critically and suggest some approaches that could be explored, as well as potential limitations of these approaches.

## Family Pedigree Studies

Family pedigree studies examine how the phenotype occurs in multigenerational families, the likelihood that parents will appear to pass on the trait to their children, and occasionally the association of genotype and phenotype. The latter is helped because families share most other genes and this reduces background genetic variability, the noise in the signal. One important conceptual limitation is a statistical one: if vasovagal syncope has a lifetime cumulative incidence of 35%, then if stochastic and not genetic there will be families with more than one fainting member, and the apparent inheritance pattern will be autosomal dominant. Simply reporting aggregated family histories cannot address this completely.

### Phenotypic Pedigrees

Kleinknecht and Lenz (7) reported that 66% of people who fainted during exposure to blood or injury had at least one fainting parent compared to only 41% of control subjects (*p* < 0.01). In a related study (8) Kleinknecht et al. reported that 94% of subjects with syncope in the absence of aversion to medical situations also had a family history of syncope. Marquez et al. (9) and Newton et al. (10, 11) have documented multigenerational pedigrees of families with numerous fainting members. The patterns were compatible with incomplete penetrance of an autosomal dominant pattern.

### Ensemble Family Histories

With a lifetime cumulative incidence of 25–40% many families would be expected to have multiple fainting members by chance alone. Pooling family data is one approach to estimate the risk conferred by family histories on its members. Mathias et al. (12, 13) reported that 36–51% of fainting patients had a family members who fainted, compared to 28% of controls. Camfield and Camfield (14) reported an apparently autosomal dominance pattern the families who had children in school. Almost all fainting children had close family members who fainted, while only 33% of non-fainting probands had a family members who fainted. In contrast, Newton et al. (10) reported that fainting probands had only a 19% likelihood of fainting family members.

These studies have a number of limitations. Most had patients as index cases, raising the possibility of inclusion bias. Few had controls and or reproducible diagnostic criteria. None featured actuarial analysis, which is important in a syndrome in which the likelihood of fainting at least once increases with age. Finally, recall bias may be common, particularly in family histories of fainting.

We reported a community-based study of family histories of vasovagal syncope (2), featuring consenting second-year medical students and their first-degree relatives. The diagnosis of vasovagal syncope was confirmed with the Calgary Syncope Score, which has been used repeatedly and successfully in randomized clinical trials. By age 60 the likelihood of syncope was estimated to be 37%, with 42% of females and 31% of males fainting. The likelihood of offspring fainting depended significantly on whether their parents fainted. For example, a man with two fainting parents is nearly 8-fold more likely to faint than one neither of whose parents faint. Therefore, the likelihood of an individual fainting depends significantly on both sex and whether and how many parents faint. This does not prove that fainting is due to genes alone, and there almost certainly are substantial environmental factors.

## Twin Studies

Twins provide an interesting opportunity for efficient assessment of genetic sources of phenotypic traits. They are siblings, and therefore share not only much of their genetic information but also usually their environmental, social, educational, and financial background. Furthermore, they provide a unique experiment in nature of gene dosing: monozygotic genes share all their genetic information while non-identical twins share only half their genes.

Marquez et al. (9) and Arikan et al. (15) reported three sets of monozygotic twins with recurrent vasovagal syncope. One pair of female twins had fainting family members while the male twins did not. One set of monozygotic male twins both had documented asystole during syncope. Klein and Berkovic performed a much larger same-sex twins study (16) in which 51 sets of twins were recruited through the Australian Twins Registry, and in which at least one subject had fainted. The 19 identical (monozygous) twin pairs had significantly higher concordance in their histories of syncope, and 7 of these pairs had multiple affected family members. Taken together the twins data are compatible with a genetic association with vasovagal syncope, but far from establish its reality.

## Candidate Gene Analyses

A candidate gene analysis tests a specific hypothesis: does a particular allele associate significantly with a specific phenotypic trait? Typically a population of patients with the abnormal phenotype is compared to a control group. These have been very popular studies but are prone to several problems. They are univariable analyses, and usually do not include other and possibly important genotypic or phenotypic information. Candidate gene association studies by their nature study only the families at hand, making them are susceptible to local imbalances between the fainting and control groups, and external validity concerns when comparing the study population to different populations. Replication is critical (17). The generally small sample sizes mean that only relatively common alleles can be studied, with a minor allele frequency usually exceeding 10%. Finally there is a persistent concern here as elsewhere about publication bias: well-done but negative studies struggle to find a home.

Most candidate gene studies of vasovagal syncope compared patients with positive tilt tests to those with negative tilt tests; in essence, a surrogate biomarker. We took a complementary approach by directly testing the association of 12 candidate gene alleles with clinical vasovagal syncope in 7 kindreds (18). The advantages of this approach are that we are studying clinical presentations and not surrogate markers, and kindred studies reduce the background variability in both genetic and environmental influences. We performed a candidate gene association study of 12 allelic variants with plausible connections to vasovagal syncope. The candidate alleles were targeted based on genetic and physiologic plausibility, and vasovagal syncope was ascertained with a validated questionnaire. One insertion/deletion promoter variant and 11 SNPs, including 2 promoter variants, were selected because they had a known physiologic effect, were plausibly relevant to vasovagal syncope physiology, and had a minor allelic frequency of at least 15%. This frequency was chosen based on the results of an earlier family history study and a Mendelian model of autosomal dominance (2).

In this Candidate Gene section we'll review studies of vasovagal syncope and other disorders of hemodynamic control, using both surrogate studies and direct comparisons of patients and asymptomatic control subjects. Table 1 contains a compilation attempts to demonstrate associations between specific candidate genes and the susceptibility to the induction of vasovagal syncope on tilt tests.

**Table 1:** Associations between candidate gene alleles and susceptibility to induction of vasovagal syncope on tilt table tests.

**Gene**	**Protein**	**Genotype-phenotype association**
ACE	Angiotensin converting enzyme	Two negative (19, 20)
AGT	Angiotensinogen	Negative (20)
ATR1	Angiotensin 2 receptor	Negative (20)
EDN1	Endothelin 1	Positive (21), negative clinical
EDNRA	Endothelin type A receptor	Negative (21)
GNAS1	G protein alpha	One positive (22), one negative (23)
GNB3	G protein Beta3	One positive (24), four negative (22, 23, 25, 26)
GNG2	G protein γ2 subunit	Negative (27)
RGS2	G protein signaling regulator	Three negative (22, 23, 28)
ADRA1A	Alpha1 adrenergic receptor	One negative (25), one positive (29)
ADRB1	Beta1 adrenergic receptor	Two positive (29, 30), two negative (25, 31)
ADRB2	Beta2 adrenergic receptor	Negative (25)
ADORA2A	Adenosine receptor A2A	Positive (32)
SERT	Serotonin transporter	Two negative (20, 25)
DBH	Dopamine beta hydroxylase	Negative (25)
CHRM2	Muscarinic M2 receptor	Negative (27)
KCNJ3	Potassium inwardly rectifying channel, subfamily J, member 3	Negative (27)
KCNJ5	Potassium inwardly rectifying channel, subfamily J, member 5	Negative (27)

### Alpha Adrenergic Receptors

The *alpha adrenergic 1 receptor* ADRA1A 1039T>C variant is involved in sympathetic transduction, and the Arg/Arg genotype of *ADRA1A* 1039 T>C significantly associated with positive tilt tests when comparing vasovagal syncope patients to asymptomatic controls with negative tilt tests (29). However, it was unclear if the clinical variable was the vasovagal syncope phenotype or the positive tilt test. We found no evidence that this allele associates with vasovagal syncope subjects compared to unaffected family members (18). Our results suggest that the ADRA1A variant associates with positive tilt tests, but not with vasovagal syncope.

### Beta Adrenergic Receptors

The *beta adrenergic receptor* ADRB1 1165G>C (30) and 145A>G (25) variants are involved in sympathetic transduction, might be involved in the vasovagal reflex. The genotypes of the *beta adrenergic receptor gene ADRB1 1165G*>*C* associated with positive tilt tests in syncope patients (30). However, the control population was syncope patients with negative tilt responses, not unaffected controls. Furthermore, neither we (18) nor others (25, 31) detected an association of the ADRB1 1165G>C alleles with the clinical vasovagal phenotype when compared with unaffected control subjects.

Marquez et al. (30) studied the β*1-adrenoceptor polymorphism (Gly389Arg)* in 50 syncope patients who had tilt tests. These polymorphisms were studied because of the role of beta adrenergic stimulation in inducing vasovagal syncope during tilt testing (33), and because the 389Arg allele may increase sensitivity to β-adrenergic stimulation (34). The results were in complete contrast to the apparent hypothesis, and in fact the allele was equally prevalent in syncope patients and in larger normal populations. In the candidate gene kindred analysis (18) there was no evidence for the association of either allele with vasovagal syncope. Therefore, the Arg389Gly allele of the β1-adrenoceptor may make a positive tilt test more likely but does not seem to predispose to clinical vasovagal syncope.

### G Protein Signaling

The beta-adrenergic G alpha subunit GNAS1 351C>T variant (35) is involved in sympathetic transduction, and is linked to orthostatic intolerance. We found no evidence that this allele associates with vasovagal syncope subjects compared to unaffected family members. Our results suggest that the ADRA1A variant is not associated with vasovagal syncope. Similarly, the TT genotype of GNAS1 T393C associates with less hypotension in patients with orthostatic hypotension (35), but not with tilt test outcome in patients with presumed vasovagal syncope (23). Finally, Lelonek et al. reported that 825TT genotype of GNB3, the G protein beta 3 subunit predicts negative tilt test result in syncope patients (23). We did not test this genotype. They also reported the association of the 825T allele with atypical histories of vasovagal syncope.

### Adenosine Receptors

The *adenosine 2A receptor ADORA2A 1083T*>*C (formerly 1364 T*>*C)* variant (32) is involved in adenosine transduction, and is linked to orthostatic hypotension and bradycardia. The C/C genotype of the *adenosine receptor ADORA2A 1083T*>*C* associates with positive tilt tests (32). We found no evidence that this allele associates with vasovagal syncope subjects compared to unaffected family members (18). Our results indicate that the ADORA2A variant associates with positive tilt tests, but not with vasovagal syncope.

### Vasoactive Receptors

Newton et al. (19) reported that a polymorphism in the angiotensin-converting enzyme was approximately equally prevalent in syncope patients with a positive tilt test and in the normative population, making it an unlikely candidate for a gene that causes vasovagal syncope. Arterial vasoconstriction is impaired in young vasovagal syncope patients, and that this is prevented by NOS inhibition (36). eNOS3 (−786)T>C and 894T>G variants associate with Postural Tachycardia Syndrome (37). However, we found no evidence that this allele associates with vasovagal syncope subjects compared to unaffected family members (18).

### Serotonin Signaling

We identified 3 gene variants in serotonin and dopamine signaling that appear to be associated with the phenotype of vasovagal syncope. The serotonin 5HT1A receptor is linked to vasodilation, might be involved in the vasovagal reflex (38–40), and the (-1019) G>C promoter variants regulate receptor levels. The serotonin reuptake transporter long/short promoter variant modulates serotonin signaling, and is linked to positive tilt test phenotype (41). Catecholamine O-methyltransferase degrades dopamine, and thereby reduces serotonin release into the synapse (42, 43). The serotonin 5HT1A receptor (-1019) G allele strongly associates with syncope in males but has the opposite effect females (*p* = 0.005). The serotonin transporter promoter long alleles associate with a decreased likelihood of fainting in males but increased in females. Males with homozygous long and short promoter alleles had 25 and 47% likelihoods of fainting, respectively, while in females had the likelihoods were 75 and 50%. The catechol O-methyltransferase c.472 A alleles were significantly linked with a decreased likelihood of fainting in males but the opposite in females (*p* = 0.017). This effect of serotonin signaling alleles on vasovagal syncope supports the serotonin hypothesis of the physiology of vasovagal syncope.

### Candidate Genes and Serotonin Model

These findings support a model in which serotonin binding to post-synaptic 5HT1A receptors causes vasovagal syncope by inducing hypotension and bradycardia (40, 41). The possible interactions of serotonin signaling and vasovagal syncope have been noted for decades. One of the clearest series studies demonstrated that acute intravenous administration of clomipramine, a highly specific serotonin transporter inhibitor, provoked vasovagal syncope on tilt tests (41, 44, 45). Therefore, acutely increased intrasynaptic serotonin is associated with the vasovagal response. Randomized clinical trials of serotonin transport inhibitors have provided mixed results (46–48), but two good studies have been positive. Our work does provide one conceptual framework for this field, but much uncertainty remains.

## Genome-Wide Association Studies

Genome-wide association studies, or GWAS, rely on the presence of millions of single nucleotide polymorphisms, or SNPs, scattered in the genome, all of whose positions on chromosomes are known. Studies search for highly significant statistical significance between specific SNPs and the phenotypes of interest. Generally because so many SNPs are studied simultaneously the threshold for statistical significance is extraordinarily high, often 10^−8^ or higher. As well, GWAS studies usually require replication, and usually thousands or tens of thousands of subjects are required in both the trait and control populations. It is not uncommon to have very many SNPs have phenotype associations with statistical significances of 10^−6^. Although initially this was thought to be simply generated by the very large number of SNPs being studied this might not be so. The understanding that phenotypes arise from networks of gene and protein interactions has led to the concept of omnigenics (49), which proposes that very high numbers of genes underlie traits. In this paradigm GWAS studies target only the most influential of many genes.

### Copy Number Variants

Copy number variants are contiguous sequences of several 100 kilobases that occur in more than 2 copies per genome. Demir et al. (50) studied the distribution of copy number variants throughout the genome of 16 subjects with vasovagal syncope and 3 controls, all within 4 families. In this small study there were 26 copy number variants whose distributions varied between syncope subjects, controls, and a publicly referenced database. The interpretation was made difficult because the variants occurred on all chromosomes, the distributions differed among all subjects, some increased in number while others decreased, and some had longer contiguous stretches while others decreased in length.

### Biobank GWAS

Hadji-Turdeghal et al. (51) attempted to use GWAS to identify a genetic locus for syncope. They studied very large biobanks and databases in the United Kingdom and Denmark. The Danish population had a majority of patients with at least one of six major mental illnesses such as autism and schizophrenia. Only one locus, at chromosomal location 2q32.1, was highly significantly associated with identified syncope. The nearest known structural gene was ZNF804A, which codes for a zinc finger regulatory protein. However, only 2.2% of the UK population and 6.5% of the Danish population were identified as having syncope, suggesting that syncope was under-reported in the control populations. The biological relevance of this is tantalizing but its clinical reality and significance are yet to be determined.

### Single Family GWAS

Klein et al. (52) studied 44 Australian families with a familial history of syncope and identified 6 families with apparent autosomal dominant inheritance. Microsatellite markers identified in the largest family an apparent locus in chromosome locus 15q26, but sequencing of nearby candidate genes did not reveal mutations. Four affected members in this family did not carry the 15q26 haplotype, and the two next largest affected families did not carry this haplotype either. The relevance of this isolated finding remains to be determined.

## Future Perspectives

The evidence to date is compatible with one or more genetic sources of the vasovagal reflex. However, the studies to date are plagued by incomplete case finding, inadequate and irreproducible case definitions, limited definition of what is normal, low sample sizes, the use of surrogate outcomes that have not been substantiated in clinical studies, and the predominance of univariable candidate gene analyses. It may also be that the omnigenic or heavily polygenic paradigms are the case, and numerous genes each with small effects combine to predispose people to the vasovagal reflex. However, if there is a small number of genetic loci involved then the following criteria should be met to decide that a genetic locus is importantly involved.

1. The diagnosis should be established firmly in subjects with syncope by an evidence-based history or tilt test or documented vital signs during a syncopal spell;
2. The core abnormal allele should be present in all subjects with vasovagal syncope;
3. The core abnormal allele should be absent in most subjects without vasovagal syncope;
4. The difference between subjects with vasovagal syncope and controls should be based on clinical history and not a surrogate outcome such as a tilt table test;
5. Control subjects should be at least 50 years old, given that most (but not all) subjects with vasovagal syncope have a first syncopal spell before age 50;
6. The genetic locus should be associated with syncope in both sexes and in multiple ethnic populations;
7. There may be modifying loci that differ between males and females;
8. The findings must be reproducible in adequately powered studies.

## Author Contributions

All authors listed have made a substantial, direct and intellectual contribution to the work, and approved it for publication.

### Conflict of Interest

The authors declare that the research was conducted in the absence of any commercial or financial relationships that could be construed as a potential conflict of interest.
